# Sphingosine 1-phosphate levels in cerebrospinal fluid after subarachnoid hemorrhage

**DOI:** 10.1186/s42466-020-00093-x

**Published:** 2020-11-23

**Authors:** Anika Männer, Dominique Thomas, Marlies Wagner, Jürgen Konczalla, Helmuth Steinmetz, Robert Brunkhorst, Waltraud Pfeilschifter

**Affiliations:** 1 Frankfurt University Hospital, Department of Neurology, Goethe University, Theodor-Stern-Kai 7, 60590 Frankfurt am Main, Germany; 2 Pharmazentrum Frankfurt, Frankfurt University Hospital, Department of Clinical Pharmacology, Goethe University, Theodor-Stern-Kai 7, 60590 Frankfurt am Main, Germany; 3 Frankfurt University Hospital, Institute for Diagnostic and Interventional Neuroradiology, Goethe University, Theodor-Stern-Kai 7, 60590 Frankfurt am Main, Germany; 4 Frankfurt University Hospital, Department of Neurosurgery, Goethe University, Theodor-Stern-Kai 7, 60590 Frankfurt am Main, Germany; 5 RWTH Uniklinik Aachen, Klinik für Neurologie, Pauwelsstraße 30, 52074 Aachen, Germany

**Keywords:** Subarachnoid hemorrhage, Vasospasm, Delayed cerebral ischemia, Sphingosine 1-phosphate, Fingolimod, Neurological outcome

## Abstract

**Background and purpose:**

Sphingosin-1-phosphate (S1P) plays a crucial role as a signaling molecule in the immune system and the vasculature. Previous studies suggested a role as a vasoconstrictor of cerebral arteries via the S1P3-Receptor. Cerebral vasospasm (VS) following aneurysmal subarachnoid hemorrhage (SAH) is a major cause of disability and poor neurological outcome. Early detection of vasospasm could facilitate the prevention of cerebral ischemia in SAH patients. The aim of this prospective case-control study was to characterize the dynamics of S1P in the cerebrospinal fluid (CSF) of patients with SAH in relation to hemorrhage volume, the occurrence of VS, and neurological outcome.

**Methods:**

S1P levels in CSF of 18 control subjects and 18 SAH patients with placement of an external ventricular drainage (EVD) were determined by high sensitivity mass spectrometry from day 1 through 14 after SAH onset. Hemorrhage volume, development of asymptomatic vasospasm (aVS) and symptomatic vasospasm (sVS), and neurological outcome were correlated to day 1 S1P levels.

**Results:**

The intrathecal S1P levels of SAH patients were higher than those of the control subjects, and correlated with hemorrhage volume. There was no significant difference in S1P levels between patients with aVS and those with sVS. S1P levels significantly correlated with neurological outcome on a sliding modified Rankin scale.

**Conclusion:**

S1P levels were highest directly after placement of the EVD and correlated strongly with hemorrhage volume, which may be caused by the intrathecal clot and subsequent lysis of red blood cells, an important source of S1P. We did not detect a second peak of S1P release over the course of the intensive care period.

## Introduction

Aneurysmal subarachnoid hemorrhage (SAH), accounting for approximately 5% of all strokes is a severe disease which is associated with a variety of primary and secondary complications [1]. Despite significant advances of neurointensive care, patient outcome remains poor in many cases. Only about half of the patients (55%) who survive the initial bleed recover functional independence in most activities of daily living after SAH [2]. Particularly the development of vasospasm (VS) and delayed cerebral ischemia (DCI) are strongly associated with poor functional outcome [3, 4]. According to Frontera et al., symptomatic vasospasm (sVS) was defined as worsening of clinical symptoms (new neurological signs or deterioration in level of consciousness) due to cerebral ischemia attributable to VS. [5] It is important to note that there are many other causes of clinical deterioration besides VS, such as infection, seizures, hypoxia, metabolic derangement, hydrocephalus, and others. Therefore, in 2010 a consensus statement by a multidisciplinary group proposed a new definition for DCI and cerebral infarction after SAH [6], recommending a diagnosis of DCI only if the aforementioned other causes can be ruled out (by intensive clinical assessment, CT or MRI scan and appropriate laboratory examinations), and if the clinical deterioration persists for at least 1 h and does not appear immediately after aneurysm occlusion. Based on these hard criteria, the development of DCI is one of the most feared complications of SAH that can cause severe disability or even death [7]. Since many patients after severe SAH are comatose or sedated, the diagnosis of sVS and DCI is even more reliable if based on neuroimaging findings (CT or MRI).

Several clinical and imaging predictors of DCI after SAH have been identified, among them hemorrhage volume as graded on brain imaging with the modified Fisher score (mFS) [8]. The causative mechanisms for VS and DCI are yet unknown and there is no validated biomarker to identify patients at a high risk of VS and DCI after SAH.

Sphingosin 1-phosphate (S1P), a bioactive sphingolipid and signaling molecule, is involved in a wide variety of physiological and pathological processes, foremost in the immune system and the vasculature [9, 10]. Its plasma levels are in the low micromolar range with red blood cells being the main source of plasma S1P, whereas its concentration in interstitial fluids is much lower [10]. Via this gradient, S1P regulates lymphocyte egress from the lymph nodes. The S1P pathway can be targeted pharmacologically in humans: the synthetic S1P analog FTY720 (fingolimod) has been marketed in 2010 as the first oral drug to prevent relapses in multiple sclerosis. Its presumed mechanisms of action are a reduction of circulating lymphocates but also direct brain specific effects [11]. There is evidence from preclinical studies that S1P is produced in the periinfarct cortex after cerebral ischemia [12], and preclinical studies as well as small clinical trials have shown neuroprotective effects of the functional S1P receptor antagonist fingolimod in ischemic stroke [13, 14]. In an experimental model of SAH, fingolimod has been shown to reduce microvascular dysfunction and inflammation and improve neurological outcome of the animals [15]. Mechanistically, S1P has been proposed as putative mediator of VS as it has been shown to induce constriction of basilar arteries ex vivo via the S1P3 receptor [16]. S1P levels in CSF are low in healthy subjects (< 5 nM) and significantly elevated in early relapsing-remitting multiple sclerosis or infectious CNS diseases [17, 18]. After SAH, the lysis of red blood cells in SAH could lead to elevated S1P levels which in turn could contribute to VS and DCI.

The aim of this prospective case-control study was to characterize the dynamics of S1P levels in CSF of SAH patients over 14 days after symptom onset by high sensitivity mass spectrometry and to correlate them to hemorrhage volume, the occurrence of vasospasms and neurological outcome.

## Methods

### Patients and clinical assessment

The study was approved by the ethics committee of Frankfurt University Hospital (342/15). All consecutive SAH patients treated in our interdisciplinary neurointensive care unit from June 2016 to August 2017 were screened and informed consent was sought from patients or their legal representatives. We included patients ≥18 years of age with nontraumatic aneurysmal SAH requiring an external ventricular drain (EVD) in the early management of hydrocephalus and elevated intracranial pressure. Exclusion criteria were pregnancy or previous neurological disorders (modified Rankin Scale [mRS] ≥ 2). Samples of cerebrospinal fluid (CSF) were obtained from the EVD daily from day 1 to 14.

Clinical outcome was determined at 12 month after discharge (mRS) (Table 1). Control subjects (*n* = 18) were nonpregnant patients ≥18 years of age who underwent lumbar puncture for the exclusion of SAH or inflammatory central nervous system (CNS) diseases and had normal CSF analysis.

**Table 1:** Clinical characteristics and neurointensive care management

Patient	Age (years), Sex	Location of Aneurysm	Hemorrhage on initial CT graded on modified Fisher scale [1]	Hunt & Hess grade	WFNS grade	Initial GCS	Treatment	Onset of VS (day)	Confirmation of VS (CTA, DSA, MRI)	Typef VS	Detection of Infarction (CT/MRI)	MRI	Outcome after 1 year (mRS)
1	55, female	pericallosal artery	4	V	5	S/I/V (3^a^)	Coiling	6	CTA	aVS	-	no	4
2	38, female	posterior communicating artery	3	III	4	11	Clipping	5	DSA, CTA	aVS	-	yes	2
3	67, male	perimesencephalic SAH, no aneurysm	3	III	3	15	no aneurysma found	-	-	nVS	-	no	0
4	68, male	basilar artery	4	V	5	5	Coiling	9	CTA	sVS	CT	no	5
5	45, male	cortical SAH, no aneurysm	1	III	2	14	no aneurysm found	1	-	aVS	-	yes	0
6	41, male	middle cerebral artery	3	IV	5	S/I/V (6^a^)	Clipping	1	DSA	aVS	-	no	1
7	84, male	anterior communicating artery	4	III	4	11	Coiling	8	CTA	sVS	CT	no	5
8	71, female	posterior communicating artery	4	IV	4	S/I/V (8^a^)	Coiling	2	DSA, CTA	sVS	CT, MRI	yes	5
9	61, female	middle cerebral artery	4	V	5	S/I/V (3^a^)	Clipping	8	DSA, CTA	sVS	CT	no	4
10	60, female	vertebral artery	3	I	1	15	Coiling	2		aVS	-	no	0
11	50, male	anterior communicating artery	3	II	2	13	Clipping	4	DSA, CTA	aVS	-	no	0
12	53, male	basilar artery	4	V	5	S/I/V (3^a^)	Coiling	7	CTA	sVS	CT, MRI	yes	4
13	66, female	anterior communicating artery	4	IV	4	11	Coiling	11	CTA, MRA	sVS	CT, MRI	yes	3
14	71, female	anterior communicating artery	4	V	5	S/I/V (5^a^)	Clipping	1	DSA, CTA	aVS	-	no	6
15	46, female	posterior communicating artery	4	IV	4	10	Clipping	-	CTA	nVS	-	yes	4
16	61, male	anterior communicating artery	1	III	2	14	Clipping	6	CTA	aVS	-	no	0
17	34, male	basilar artery	4	IV	5	S/I/V (4^a^)	Coiling	6	-	aVS	-	yes	2
18	53, female	anterior communicating artery	4	III	3	13	Coiling	3	CTA	sVS	CT	no	5

*VS* vasospasms, *aVS* asymptomatic vasospasms: evidence of vessel narrowing on CTA or Doppler sonography without cerebral ischemia, *sVS* symptomatic vasospasms: evidence of vessel narrowing on CTA or Doppler sonography with brain infarcts on CT or MRI, *nVS* no vasospasms: no evidence of vessel narrowing, ^a^ - GCS as documented prior to sedation/intubation by EMS

Aneurysms were detected by digital subtraction catheter angiography (DSA). According to the assessment of an interdisciplinary neurovascular team, half of the patients were treated by occlusion of the aneurysm by neurosurgical clipping (*n* = 8), the other half of the patients were treated by endovascular occlusion by coiling (n = 8) (Table 1). All patients had aneurysm repair within the first 24 h after admission to the hospital and received the standard therapy for SAH in accordance to national and international guidelines: oral nimodipine (360 mg/day), close monitoring of the mean arterial pressure (MAP), and the use of catecholamines to maintain a MAP of 60–90 mmHg prior to detection of VS and > 100 mg - if tolerated - after sonographic detection of VS.

Head CT was performed upon admission (in all cases within 24 h after symptom onset) and between 24 and 48 h after surgical or endovascular aneurysm repair (to examine the development of edema, secondary bleeding or new cerebral infarction) and before discharge. Additional brain imaging (CT mostly with CT angiography or MR with MR angiography) were performed as required when patients showed a clinical deterioration.

The development of vasospasm was monitored routinely by daily transcranial Doppler (TCD) ultrasonography. TCD vasospasm was defined as a mean flow velocity in the middle cerebral artery (MCA) ≥ 120 cm/sec [19]. In cases of vasospasm on TCD, conservative management was supplemented by induced hypertension (MAP > 100 mmHg). In most cases, vasospasm was confirmed by CT or MR angiography or digital subtraction angiography (DSA) (defined by > 1/3 vasospastic arterial narrowing not attributable to atherosclerosis, catheter-induced spasm or vessel hypoplasia).

Because our patient cohort comprised mainly analgosedated patiens whose neurological function in terms of focal deficits could not be reliably assessed, we applied a strict definition of sVS: the documented vasospasm had to be compatible with new cerebral ischemia on clinically-indicated follow up brain imaging (28% MRI, 72% CT) that had not been present on the imaging directly after aneurysm occlusion. Other causes of cerebral ischemia or DCI had to be excluded (re-bleeding, hydrocephalus and others).

### Determination of sphingolipid concentrations by liquid chromatography/tandem mass spectrometry (LC-MS/MS)

CSF samples were centrifuged at 1000×g for 5 min at 4 °C and the supernatant stored at − 80 °C until analysis. CSF samples (100 μL) were mixed with 100 μL buffer (citric acid 30 mM, disodium hydrogen phosphate 40 mM) and 20 μL internal standard solution containing sphingosine-d7, sphinganine-d7 (30 ng/mL each), sphingosine-1-phosphate-d7 (40 ng/mL, avanti polar lipids, Alabaster, USA). The mixture was extracted with 600 μL methanol/chloroform/hydrochloric acid (15:83:2, v/v/v). The organic phase was evaporated and reconstituted in 50 μL of methanol.

Analytes were separated using an Agilent 1260 series binary pump (Agilent technologies, Waldbronn, Germany) equipped with a Kinetex EVO C18 column (50 mm × 2.1 mm, 1.7 μm, 100 Å; Phenomenex, Aschaffenburg, Germany). Column temperature was 55 °C. Mobile phases were 0.5% formic acid and acetonitrile/isopropanol/acetone (50:30:20, v/v/v) with 1% formic acid. A gradient program was used at a flow rate of 0.3 mL/min. Total running time was 7.5 min. After every sample, ethanol was injected. The MS/MS analyses were performed in Multiple Reaction Monitoring (MRM) mode using a triple quadrupole mass spectrometer 5500QTRAP (Sciex, Darmstadt, Germany) operating in positive electrospray ionization mode.

Data Acquisition was performed using Analyst Software V 1.6 and quantification was performed with MultiQuant Software V 3.0 (Sciex, Darmstadt, Germany), employing isotope dilution mass spectrometry.

### Statistical analyses

Statistical analyses were performed with Prism 7 (GraphPad Software, La Jolla, CA) and BiAS (Biometische Analyse von Stichproben, Epsilon Editorial). Results are given as mean ± standard deviation (SD), the primary variable is the CSF-S1P concentration in ng/ml. For all tests, a *p* value of less than 0.05 was considered significant. All variables were tested for normal distribution by the Shapiro-Wilk Test and the respective statistic test method is detailed along with the results.

## Results

### Patient characteristics

Eighteen SAH patients (Table 1) were recruited and 18 control patients were matched. The two groups did not differ significantly in age or sex distribution (SAH group: mean age 57 ± 13 years, 50% females; controls: mean age 53 ± 18 years, 50% females). All SAH patients underwent aneurysm repair within 24 h of admission.

### S1P levels in CSF are elevated after SAH and correlate with hemorrhage volume

**Fig. 1:**
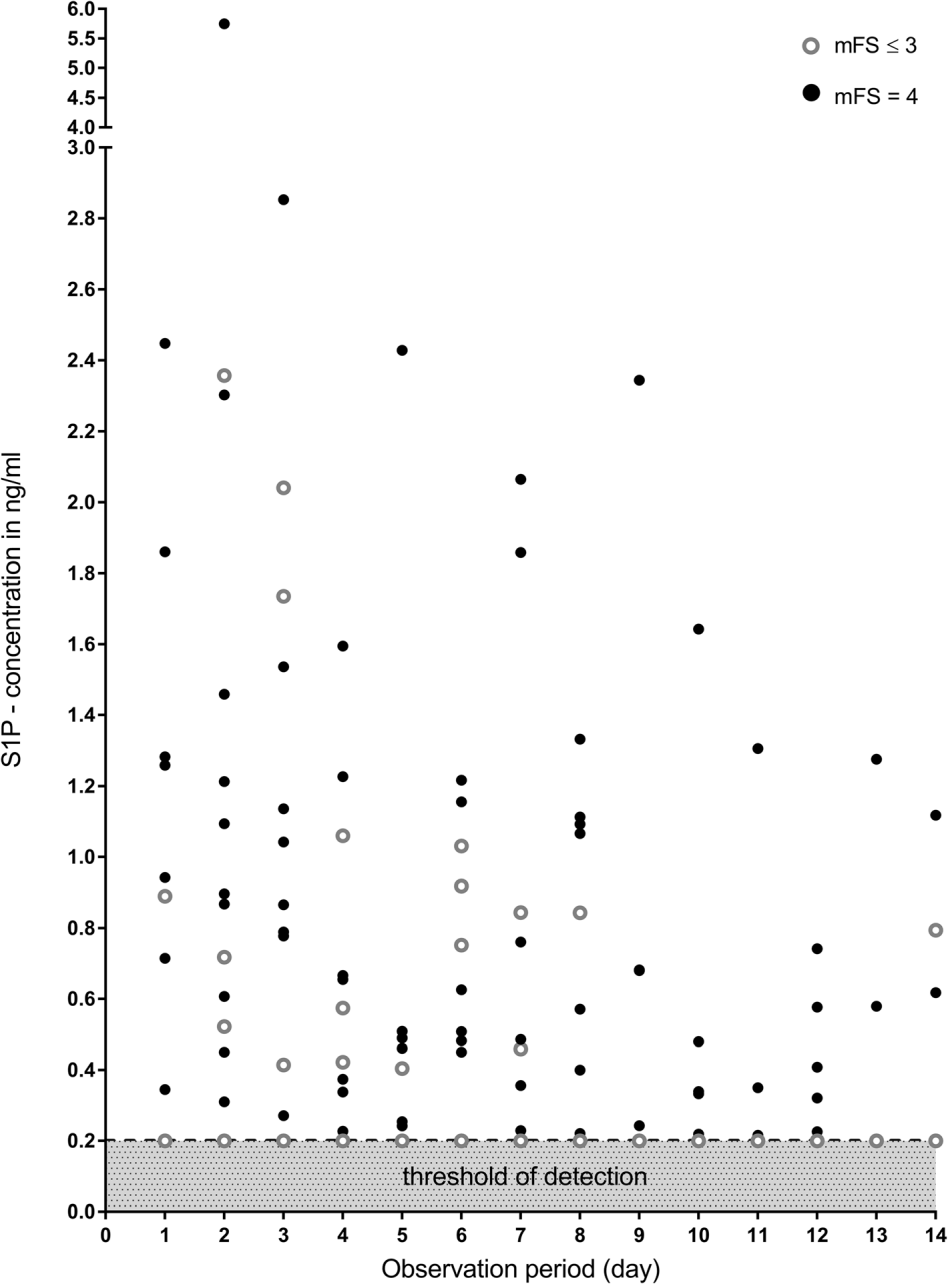
S1P levels in CSF in SAH, measured at day 1 to day 14. S1P levels in CSF were measured with high sensitivity tandem mass spectrometry. Only values above the threshold of detection (0.2 ng/ml = 0.52 nM) are depicted. S1P levels of all control patients were below the threshold of detection

**Fig. 2:**
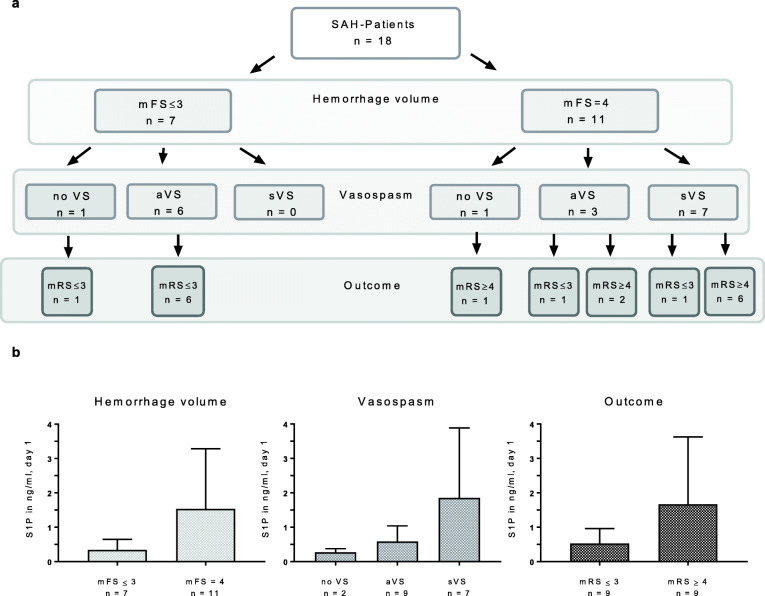
**a** Hemorrhage volume, detection of vasospasm and functional outcome at 90 days in the SAH patient cohort. mFS = modified Fisher scale, nVS =no vasospasm, aVS = asymptomatic vasospasm, sVS = symptomatic vasospasm, mRS = modified Rankin scale score. **b** Correlation of S1P levels in CSF with dichotomized hemorrhage volume, occurrence of vasospasm and neurological outcome. Bars represent the means ± SD. mFS – modified Fisher Scale, sVS – symptomatic vasospasm, aVS – asymptomatic vasospasm, mRS – modified Rankin scale score

S1P was detectable (threshold of detection ≤0.2 ng/ml or 0.53 nM) in the CSF of 9/18 patients on day 1 after SAH and in additional 3 patients up to day 10 (Fig. 1). We did not find a significant difference in S1P levels in patients with neurosurgical in comparison to endovascular treatment. In the control cohort, CSF S1P levels were below the threshold of detection, representing a significant increase after SAH (Fisher’s exact test at day 1, *p* = 0.000516) (Fig. 1). S1P levels in SAH patients were heterogeneous but showed a significant decrease after 14 days (Wilcoxon matched pairs test, Z = 2.3664, *p* = 0.017960). Fig. 2 illustrates the distribution of clinical characteristics in our patient cohort. In patients with large hemorrhage volume, indicated by modified Fisher Scale [20] (mFS) = 4 (11/18, 61%) S1P levels were significantly higher than in SAH patients with mFS ≤ 3 (Wilcoxon Mann Whitney test, Z = 2.150, *p* = 0.031528) (Fig. 2b). S1P levels at day 1 and mFS grade in the whole SAH patient cohort showed a significant correlation (Kendall Tau analysis, tau ß =0.502, *p* = 0.029, data not shown).

### Correlation of S1P levels in CSF with development of vasospasm and neurological outcome

More than 50% of our patient cohort had a large hemorrhage volume (mFS = 4) (Fig. 2a). 16/18 (88%) patients developed VS (7/16 [44%] sVS and 9/16 [56%] asymptomatic vasospasm [aVS]). Symptomatic VS only occurred in patients with large hematomas (mFS = 4). Although there was a trend towards higher S1P levels in patients with sVS, there was no significant difference between the mean S1P levels between groups with aVS and sVS (Wilcoxon Mann Whitney test, Z = 1.470, *p* = 0.141694) (Fig. 2b). The onset of VS was between as early as day 1 and as late as day 8. In the individual patients, there was no significant difference between the S1P level on day 1 and the level on the day of VS detection (Wilcoxon matched pairs test, Z = 1.3337, *p* = 0.182315, data not shown).

In a next step, S1P levels were correlated with neurological outcome at 12 months. We observed a significant correlation between the S1P level at day 1 and the mRS on a sliding scale (tau ß = 0.559, *p* = 0.007, data not shown). A dichotomized analysis of poor outcome (mRS ≥ 4) and favourable outcome (mRS ≤ 3) showed a trend of higher day 1 S1P levels in patients with poor outcome without reaching statistical significance (Wilcoxon Mann Whitney test, Z = 1.673, *p* = 0.094264) (Fig. 2b).

## Discussion

In this prospective case-control study of S1P levels in the CSF of SAH patients over 14 days, we found that contrary to healthy controls, whose S1P levels were below the threshold of detection, S1P was detectable in the low nanomolar range in half of the patients, thus representing a significant increase after SAH. Owing to the significant correlation of S1P levels with hemorrhage volume and to the temporal profile with decreasing S1P levels over the course of 14 days, we assume that the increase in intrathecal S1P can mainly be attributed to the hemorrhage and subsequent lysis of red blood cells. This was also supported by the lack of a temporal association between S1P levels and the occurrence of vasospasm or new cerebral infarction. By contrast, a secondary intrathecal production, e.g. by invading immune cells or release from ischemic brain tissue, does not seem to play a relevant role since in this case secondary increases of S1P levels would have to be expected. The significant correlation between S1P levels and neurological outcome on a sliding mRS scale could well reflect the known influence of hemorrhage volume on outcome after SAH, so that our study does not yet justify the assumption of an independent role of CSF-S1P as a predictive biomarker in SAH. Our data do not exclude that S1P contributes to VS after SAH, which would be pathophysiologically conceivable since S1P has been shown to act as a vasoconstrictor on cerebral arteries [16] and treatment of experimental animals with the functional S1P receptor antagonist fingolimod has been shown to ameliorate the sequelae of SAH [15]. Nevertheless, the relatively low CSF concentrations after SAH found in our study do not encourage e.g. the intrathecal scavenging of this mediator with a neutralizing antibody as it has been proposed for other diseases [21].

To the best of our knowledge, this is the first prospective study investigating the dynamics of S1P levels in CSF after SAH. S1P is a dynamic and instable lipid mediator and requires careful preanalytical processing [22]. Hence, in a previous investigation of sphingolipid profiles in the CSF of patients at a single time point post SAH, the S1P concentration was below the limit of detection and did not allow correlation with clinical parameters [23].

A strength of our study is the daily sampling over 14 days which allowed to characterize the temporal dynamics of intrathecal S1P after SAH and excluded a secondary increase during the VS phase but the limitation of a relatively small sample size, especially in view of the large variability of the S1P concentrations, has to be taken into account.

## Data Availability

The dataset of the current study is available from the corresponding author upon reasonable request.
